# Asparaginyl endopeptidases: enzymology, applications and limitations

**DOI:** 10.1039/d1ob00608h

**Published:** 2021-05-12

**Authors:** T. M. Simon Tang, Louis Y. P. Luk

**Affiliations:** School of Chemistry, Cardiff University Main Building Park Place Cardiff CF10 3AT UK lukly@cardiff.ac.uk; Cardiff Catalysis Institute, School of Chemistry, Cardiff University Main Building Park Place Cardiff CF10 3AT UK

## Abstract

Asparaginyl endopeptidases (AEP) are cysteine proteases found in mammalian and plant cells. Several AEP isoforms from plant species were found to exhibit transpeptidase activity which is integral for the key head-to-tail cyclisation reaction during the biosynthesis of cyclotides. Since many plant AEPs exhibit excellent enzyme kinetics for peptide ligation *via* a relatively short substrate recognition sequence, they have become appealing tools for peptide and protein modification. In this review, research focused on the enzymology of AEPs and their applications in polypeptide cyclisation and labelling will be presented. Importantly, the limitations of using AEPs and opportunities for future research and innovation will also be discussed.

## Abstract 2

The potentials of using asparaginyl endopeptidases (AEP) as tools for polypeptide modifications have been discussed.

## Graphical abstract

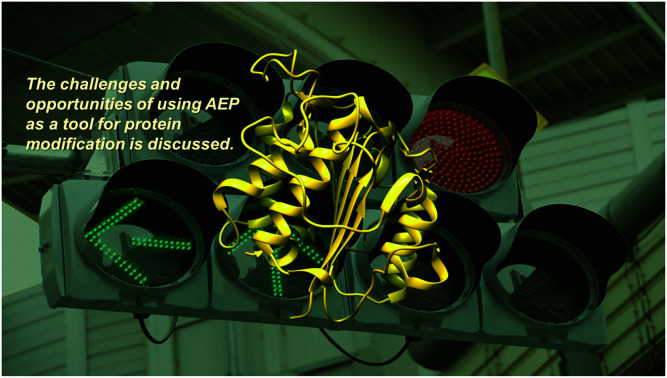

## Introduction

**Fig. 1:**
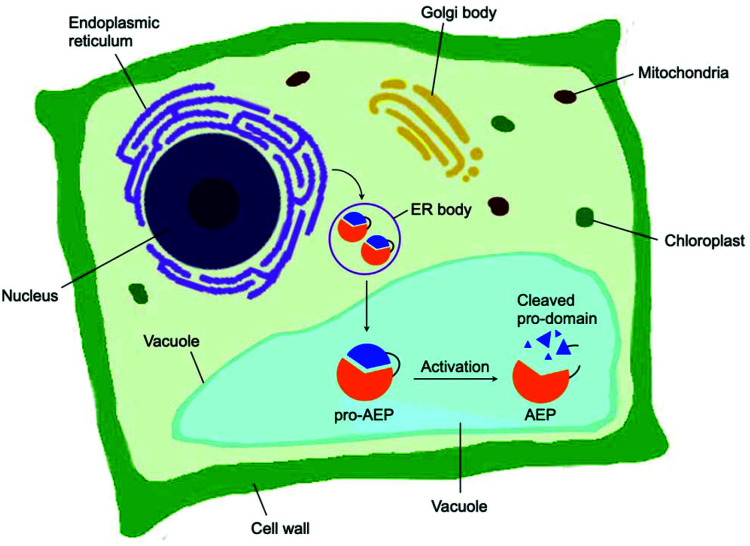
Biosynthesis of AEP in plant cells. Zymogenic AEPs are folded in the ER and stored in ER bodies. The pro-enzyme is trafficked to the vacuole, where the acidic environment enables cleavage of the pro-domain resulting in activation.

Asparaginyl endopeptidase (AEP) are proteases identified from the vacuole of plant cells.^1^ Also known as legumain,^2,3^ vacuolar processing enzyme^1^ or peptide asparaginyl ligase,^4^ AEPs react selectively at an internal asparagine (Asn) or aspartate (Asp) residue of a peptide backbone *via* a catalytic cysteine residue. In all cases, the enzymes are folded in the endoplasmic reticulum (ER) as a zymogen with a pro-domain which prevents substrate access to the active site.^5–7^ Pro-AEPs have been shown to accumulate in ER bodies within the epidermal cells of *Arabidopsis thaliana* seedlings.^5^ In response to stress, ER bodies fuse and release zymogenic AEPs into the vacuole.^5^ AEPs have also been reported to process seed storage proteins, 2S albumins, in prevacuolar multivesicular bodies (MVB) before reaching the protein storage vacuole in *A. thaliana* embryos.^8^ The acidic environment within the MVB and vacuole trigger a proteolytic maturation to afford the active AEP (Fig. 1).^2,5,7–10^

**Fig. 2:**
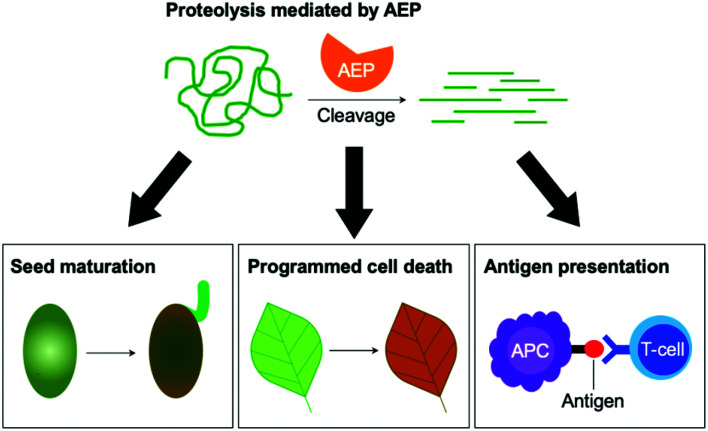
The peptidase activities of AEPs are involved in several critical cellular processes including seed maturation, programmed cell death and the regulation of antigen presentation.

Matured AEPs primarily function as peptidases which are associated with several critical cellular processes.^2,11^ Purified AEP from the seed of castor beans (*Ricinus communis*) has been shown to mediate the proteolytic processing of pro-proteins to afford mature seed storage proteins including 2S albumin and 11S globulin.^1,12^ In vegetative organs, AEPs have been shown to facilitate programmed cell death.^5,13^ When an environmental stress (concentrated salt solution) was applied to healthy seedlings of *A. thaliana*, ER bodies were found to fuse and deliver AEPs to the vacuole, leading to cell death (Fig. 2).^5^ While their structures and activity are similar to those of caspases,^14–16^ AEPs were also identified in mammalians, including humans and mice.^2,17^ Mammalian orthologues of AEPs, namely legumains, are associated with antigen processing which moderate antigen presentation and T-cell activity.^18–20^ AEP-mediated proteolytic processing of the microbial tetanus toxin antigen was found to enable antigen presentation by class II major histocompatibility complex molecules.^18^

**Fig. 3:**
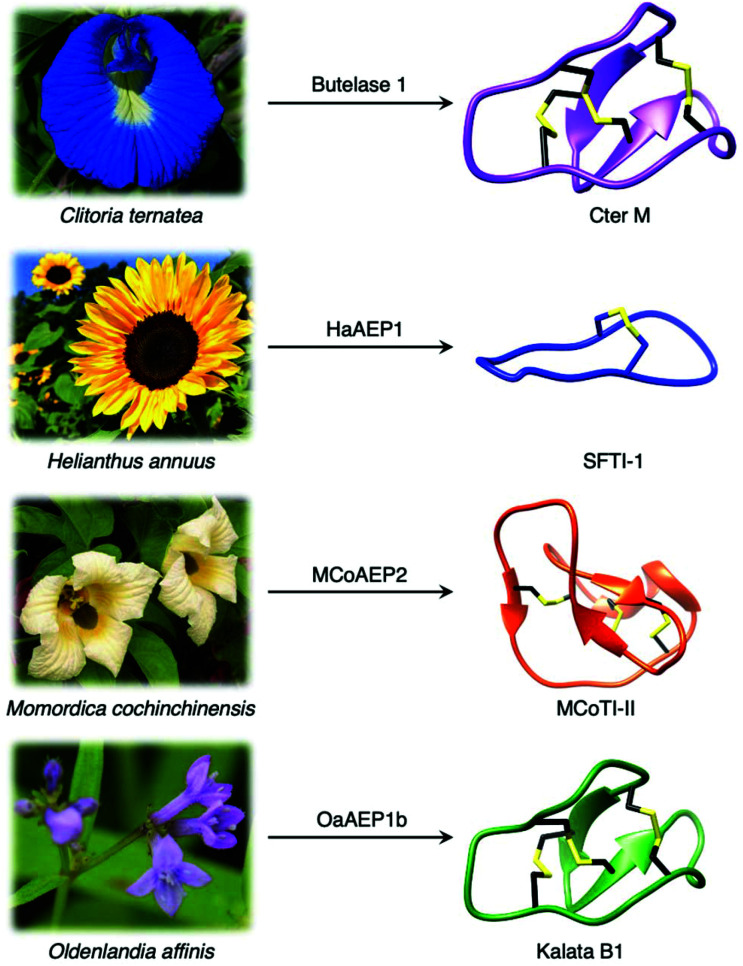
Plant species utilise AEPs with ligase activities to generate backbone cyclised peptides with enhanced stability and bioactivity. Cter M (PDB: 2LAM) from *C. ternatea*, SFTI-1 (PDB: 1JBL) from *H. annuus*, MCoTI-II (PDB: 1IB9) from *M. cochinchinensis* and kalata B1 (PDB: 1NB1) from *O. affinis*.

Although most AEPs were shown to facilitate peptide cleavage, some AEPs isolated from plant species were found to exhibit peptide ligase activity.^4,21–27^ Ligase type AEPs are rare compared to the corresponding peptidases.^4,21–23^ Various plant species have been found to utilise this peptide ligase activity to facilitate post translational modification to produce backbone cyclised peptides (Fig. 3).^4,22–24,26^ The cyclisation step during the biosynthesis of kalata B1, a cystine knotted macrocyclic peptide, was shown to be catalysed by an AEP (OaAEP1b) in *Oldenlandia affinis*.^22^ Cyclic peptides found in plants exhibit a range of bioactivities,^28,29^ and demonstrate superior stability compared to their linear counterparts.^30^ Kalata B1 extracted from *O. affinis*, a native African plant species, demonstrates insecticidal activities inhibiting the growth of *Helicoverpa punctigera* larvae at a high concentration.^31,32^ Furthermore, the cyclic kalata B1 has been shown to display high tolerance towards thermal (370 K, 97 °C) and chemical (8 M urea) denaturation.^30^ Another example of a cystine knotted cyclic peptide is Cter-M, which is cyclised by an AEP in butterfly pea plants (*Clitoria ternatea*), dubbed butelase 1.^33^ Other AEPs such as HaAEP1 from common sunflower (*Helianthus annuus*) and MCoAEP2 from gac fruit plants (*Momordica cochinchinensis*) have been reported to facilitate backbone cyclisation during the biosynthesis of cyclic trypsin inhibitor peptides SFTI-1 and MCoTI-II, respectively (Fig. 3).^27,34^

## Reaction mechanisms of AEPs

**Fig. 4:**
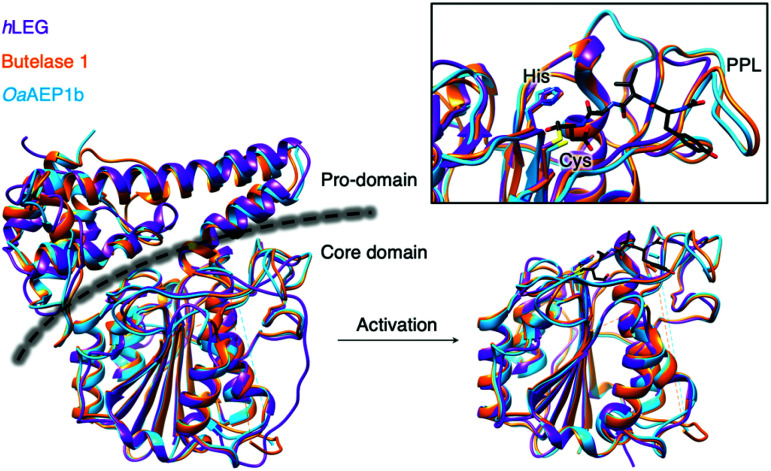
Overlaid ribbon representations of human pro-legumain (*h*LEG, PDB: 4FGU, purple), butelase 1 (PDB: 6DHI, orange), and OaAEP1b (PDB: 5H0I, blue) structures deduced from X-ray crystallography.^10,35,36^ The black dotted line indicates the separation between the pro-domain and the core domain. After activation, modified butelase 1 (PDB: 6DHI) and OaAEP1b (PDB: 5H0I) structures showing only the catalytic core domains were overlaid with the crystal structure of activated human legumain (PDB: 4AWA, purple). In the box, an enlarged image of the active sites reveals the respective catalytic diads (Cys and His), the conformation adopted by the inhibitor (YVAD-CMK) in complex with activated human legumain, and a plant specific loop region referred to as the poly-proline loop (PPL).^26^

Since the initial discovery of the AEP,^1^ there has been an expansion of research ranging from structural biology, enzymology to chemical biology development. X-ray crystallographic data of various AEPs have provided fundamental knowledge and rationales for the localisation of activity.^4,7,10,35,36^ Prominent structural features include the catalytic core domain which adopts a protein fold with a six-stranded β-sheet surrounded by five α-helices. In the proenzyme, the catalytic site is covered by the pro-domain which is C-terminal to the core domain (Fig. 4).^4,7,10,35,36^ Studies performed on an AEP from *A. thaliana* (AtLEGγ) revealed that pro-AEP adopts a dimeric state at neutral pH which has been shown to prevent enzymatic activation.^7^ The enzyme adopts a monomeric state at pH 4.0 and the protonation of salt bridges surrounding the active site of the AEP was reported to increase flexibility, enabling the auto-proteolytic cleavage of the pro-domain to generate the active enzyme.^5,7,22,35^ Taken away from the acidic and reducing environments of the plant vacuole, the cleaved core domain and pro-domain of AtLEGγ have been shown remain bound in a two-chain state.^7^ The non-covalent association of the cleaved AEP pro-domain was suggested to confer stability to the catalytic core domain at neutral pH.^7^

**Scheme 1:**
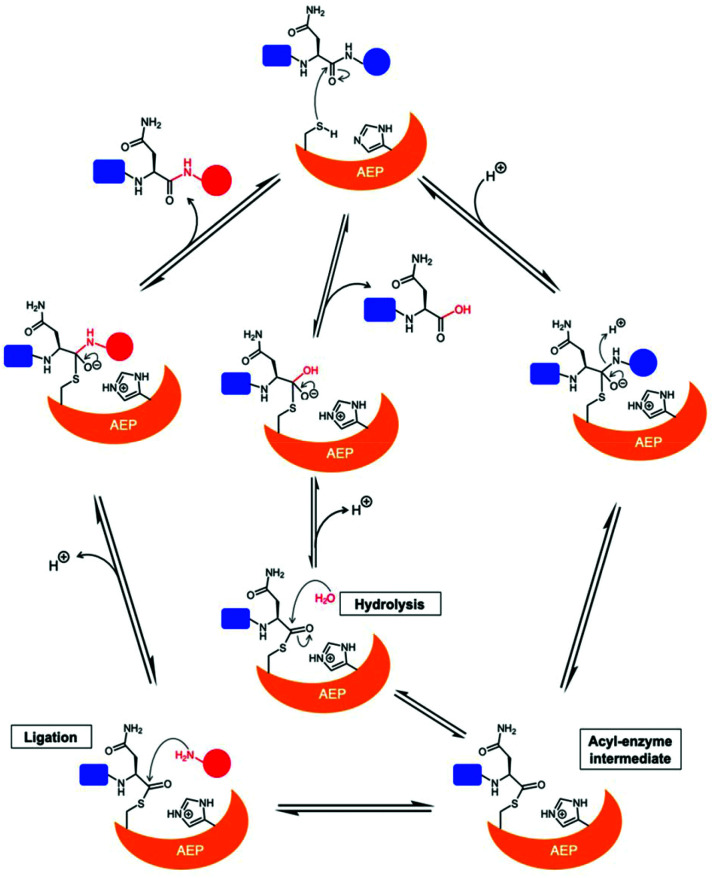
Proposed mechanism of AEPs catalysis with a Cys/His catalytic diad. AEP recognises and cleaves a peptide substrate containing Asn to form an acyl-enzyme intermediate *via* a tetrahedral intermediate. A nucleophilic attack resolves the thioester intermediate to afford the product peptide and the AEP is regenerated. The nucleophile can be a water molecule or the N-terminus of a peptide, resulting in peptide hydrolysis or ligation, respectively.

The catalytically active core domain of AEPs have been proposed to operate *via* a mechanism that recruits the catalytic cysteine residue as a nucleophile.^14,16,35^ The catalytic diad (Cys and His) mediates a nucleophilic attack on the amide carbonyl of an internal Asn residue to form a thioester acyl-enzyme intermediate (Scheme 1).^22,23,25,35^ For most AEPs, nucleophilic attack from a water molecule completes the peptide bond hydrolysis.^35^ For ligase-type AEPs, the N-terminal amine of an incoming peptide adopts the role of the nucleophile to resolve the thioester intermediate.^22,23^ The nucleophilic amine can be either from the same peptide, resulting in an intramolecular cyclisation,^22,23^ or from a different peptide, resulting in an intermolecular ligation.^23,37^ A mechanistic study of AEP-mediated peptide ligation employing ^18^O-labelled water found no isotopic shift in the ligated peptide product.^22,24^ This finding indicated that water is likely to be excluded from the active site during peptide ligation by AEPs. Peptide ligation proceeds with a direct nucleophilic attack from the N-terminal amine to the thioester intermediate with no participation of solvent water molecules.

A succinimide motif not commonly seen among other enzymes has been observed by X-ray crystallography in active sites of AEP isoforms hLEG, HaAEP1 and AtLEGγ from human, sunflower and *A. thaliana*, respectively.^25,35,38^ The metastable structure resulted from the condensation between the sidechain carboxylate of Asp and a backbone amide NH. Nevertheless, Haywood *et al.* have shown that a mutation of the succinimide forming aspartate residue to an alanine bears no influence on the ligation activity of HaAEP1.^25^

A comparison of AEPs from the same, or across multiple organisms, reveals that enzymes with ligase activities are uncommon compared to the putative proteases.^4,21–23^ Various AEP isoforms exhibit a remarkable difference in activity (protease *vs.* ligase) despite only subtle differences in sequence and structure.^4,26,35,36^ Furthermore, the activity of AEPs were reported to be influenced by the amino acid sequence, length of substrate peptides, plus reaction conditions such as pH.^22,23,39^

**Fig. 5:**
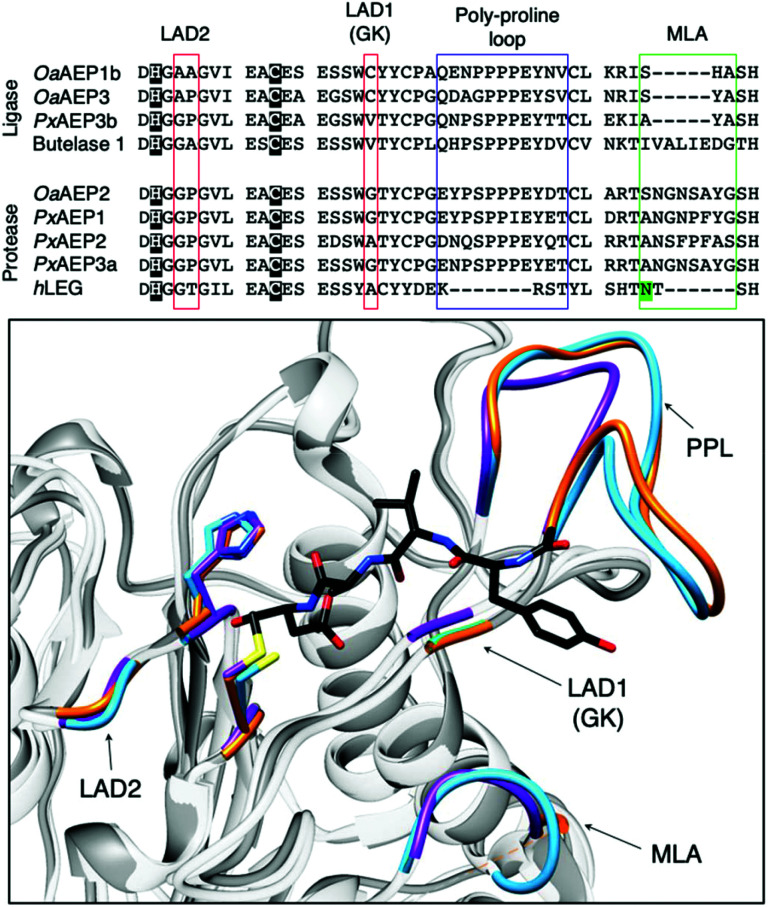
Key features associated with the determination AEP ligase and protease activity. Multiple sequence alignment of AEPs, from *O. affinis* (OaAEP1b, 3), *P. x hybrida* (PxAEP1, 2, 3a, 3b), *C. ternatea* (butelase 1) *and H. sapien* (hLEG), centred around amino acid residues reported to influence activity. Catalytic diads (Cys/His) are shown in white letters with black highlight, *N*-glycosylation site is highlighted in green, the overlaid red boxes show LAD1 (gatekeeper/GK) and LAD2, blue box shows the plant-specific poly-proline loop and the green box highlights the MLA.^4,26^ In the box, an enlarged overlaid image of OaAEP1 (PDB: 5H0I),^10^ butelase 1 (PDB: 6DHI)^36^ and human legumain (PDB: 4AWA)^35^ active sites. Key features (catalytic diad, LAD1, LAD2, PPL and MLA) are rendered blue, orange and purple for OaAEP1, butelase 1, and human legumain respectively. In black, the conformation adopted by the inhibitor (YVAD-CMK) in complex with human legumain.

The growing library of AEPs identified from numerous organisms, accompanied by the increasing availability of sequence and structural information, has enabled comparative studies among isoforms and identification of features that influence the preference towards ligase or protease activity.^7,26,27,36,39^ Substrate accessibility to the active site of AEPs are governed by a gatekeeper residue (GK), also referred to as ligase-activity determinant 1 (LAD1),^4^ has been highlighted as a factor that determines activity (Fig. 5).^10,26^ The identity of this residue, with particular emphasis on the size of its sidechain, was suggested to influence substrate access to the active site. OaAEP1b, isolated from *O. affinis*, is an AEP variant which exhibits peptide ligase activity.^22^ Mutagenesis studies of OaAEP1b demonstrated that substitutions of the gatekeeper residue (Cys247) to larger residues such as Ile, Leu and Met abolished ligase activity. In contrast, replacements with smaller Ala and Gly residues at the same position resulted in an increase in peptide cyclisation activity. In addition, the Cys247Gly mutation was shown to enhance peptide hydrolysis when compared to the wild-type and Cys247Ala variants.^10^ The smaller and less hydrophobic Gly as a gatekeeper residue was proposed to allow water molecules to access the active site, resulting in protease activity. The enhanced ligase activity of OaAEP1-C247A has consequently been adopted to facilitate protein labelling reactions in several studies.^40–42^

Further investigations into the sequence and structures of different AEP isoforms revealed additional polymorphic positions named ligase-activity determinant 2 (LAD2)^4^ and marker of ligase activity (MLA)^26^ which are located within, and adjacent to, the substrate binding pocket, respectively. The MLA was identified by a study comparing the sequences of protease and ligase AEPs from garden petunia (*Petunia x hybrida*) and describes a five amino acid deletion found in AEPs with ligase activity (Fig. 5).^26^

The impact of the MLA on the peptide cyclisation activity *in planta* was investigated using tobacco plants (*Nicotiana benthamiana*) which co-expressed transgenes encoding for AEPs and corresponding peptide substrates. Relative cyclisation efficiencies *in planta* were measured by matrix-assisted laser desorption ionisation mass spectrometry (MALDI-MS).^26^ The insertion of five amino acid to the MLA region of OaAEP1b, a putative AEP ligase, was shown to significantly impede its ability to mediate peptide cyclisation.^26^ However, when the experiments were repeated *in vitro*, the MLA modification was found to only reduce the rate of peptide cyclisation by OaAEP1b rather than a distinct shift to protease activity.^26^ The truncation described as the MLA is also not found in the amino acid sequence of butelase 1, an AEP ligase from *C. ternatea* (Fig. 5). Thus, while the MLA offer an indication to the protease *vs.* ligase preference of AEPs, it is not a critical determinant of enzyme activity.

LAD2 describes two amino acid residues lining the substrate binding pocket of AEPs. Mutation experiments performed in an AEP isolated from *Viola yedoensis* (VyPAL3) demonstrated the effect of amino acid substitutions at LAD2. Replacing Tyr with the smaller Gly residue was reported to improve the *in vitro* ligase activities of VyPAL3.^4^ Furthermore, a Tyr to Ala substitution at LAD2 of a protease AEP from *Viola canadensis* (VcAEP) was shown to shift enzyme activity from protease to ligase.^4^ However, the introduction of protease favouring residues at LAD positions did not switch the activity preference of *Vy*PAL2, an AEP ligase from *V. yedoensis*, to adapt an increase of protease activity.^4^

None of the identified structural features were found to provide absolute control over the protease *vs.* ligase preference of AEPs. Nevertheless, mutations at gatekeeper, MLA and LAD2 have all been shown to significantly impact AEP activity.^4,26^ These findings suggest that key structural features may operate synergistically to dictate AEP activity, and further investigations are required to fully establish the key determinants of AEP protease and ligase activity. A recent review by Nonis *et al.* can provide further discussions towards the key determinants of AEP protease and ligase activity.^43^

### Substrate recognition

**Scheme 2:**
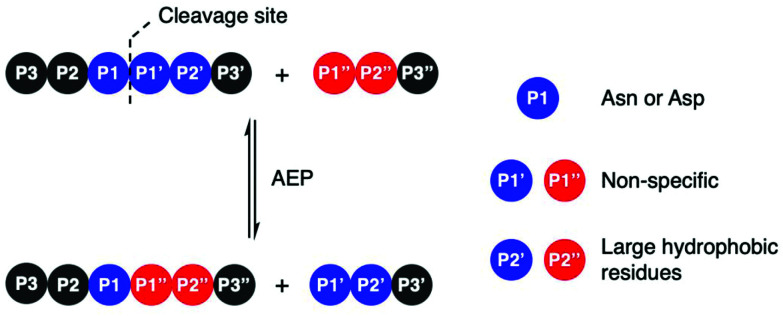
AEP-mediated transpeptidation, with amino acid residues numbered according to Schechter and Berger.^44^ The putative recognition sequences P1–P1′–P2′ and P1′′–P2′′ are coloured in blue and red, respectively.

Amino acid residues surrounding the peptide cleavage site for substrates of AEPs are commonly labelled following the numbering conventions adopted by Schechter and Berger for substrates of proteases (Scheme 2).^44^ From the cleavage site to the peptide N-terminus, amino acid residues are numbered in ascending order starting from P1. On the other side of the cleavage site, amino acid residues are denoted with a single prime and numbered in ascending order towards the C-terminus starting with P1′. For peptide ligation, the amino acid positions on the incoming peptide are labelled with a double prime and numbered in ascending order from the N-terminus starting with P1′′.

Initial studies performed with an AEP isolated from castor beans have identified strict substrate specificity towards Asn at the P1 position.^1,2,17,18,22,23^ The understanding of substrate recognition by AEPs have subsequently expanded with experiments in AEPs isolated from *C. ternatea* (butelase 1),^23,45,46^*O. affinis* (OaAEP1b and OaAEP3-5)^22,39^ and other plant species.^24,27^ Substrate recognition by AEPs were shown to be determined mostly by amino acids at positions P1, P1′ and P2′ (Scheme 2). While many AEPs have a strict preference towards Asn at P1, some isoforms such as HaAEP1 and OaAEP1b were found to accept Asp at P1.^22,24^ The preferences toward P1′ and the ensuing P1′′ from the nucleophilic peptide for ligation are non-specific. A peptide ligation assay which employed a library of peptides, with varying amino acids at the N-terminus, demonstrated that butelase 1 accepts all canonical amino acid residues at P1′′ with the exception of Pro.^23^ Furthermore, butelase 1 was reported to cyclise peptides with d-amino acids at P1′′ and recognise peptides with a thioester linkage between the P1 and P1′ residues.^45,46^ AEPs from *O. affinis* (OaAEP1b, OaAEP3-5) have been shown to process peptides with several different amino acid residues at P1′.^22,39,47^ In contrast, recognition at P2′ and P2′′ demonstrate greater specificity. While peptides with His or Ala at P2′ were not processed by OaAEP1b, peptide cyclisation was detected when substrates with Leu or Phe at P2′ were used.^22,39^ Furthermore, butelase 1 was reported to ligate peptides with large, hydrophobic side chains such as Leu, Ile and Val at P2′′.^23^

**Scheme 3:**
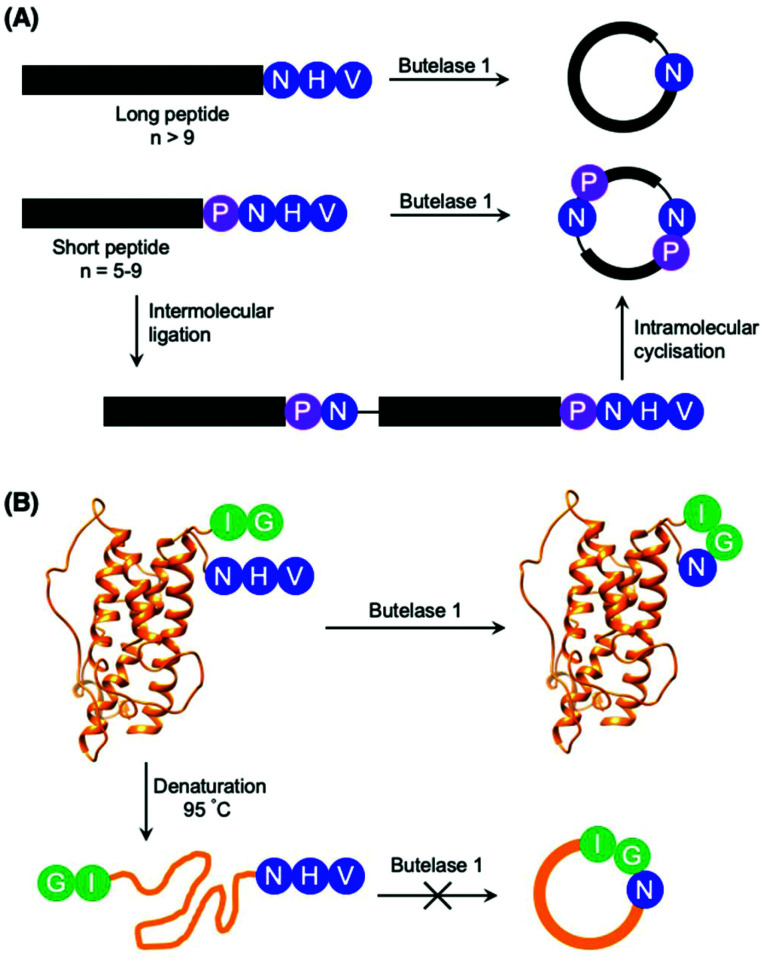
Peptide cyclisation catalysed by butelase 1. (A) Peptides greater than 9 amino acid residues in length and bearing the butelase 1 substrate recognition sequence (NHV) are cyclised by the AEP. Whereas peptides of 5–9 amino acid residues in length, with Pro at P2 and bearing the butelase 1 substrate recognition sequence (NHV) are ligated, then cyclised by AEP to afford cyclic oligomers^48^ (only cyclo-dimer shown here). (B) Butelase 1 was reported to cyclise the folded somatropin (PDB: 1HGU) but not the denatured protein.^49^

Although most investigations towards the substrate specificity of AEPs have focused on the putative recognition sequences at P1–P1′–P2′ and P1′′–P2′′ (Scheme 2), some studies suggested enzyme activity can be affected by other positions in the substrate peptide.^22,27,48^ A substitution of Arg with Ala at the P2 position was found to significantly reduce the rate of OaAEP1b-mediated peptide cleavage.^22^ Moreover, the use of Pro at P2 in a short peptide (5–9 amino acids) was shown to favour intermolecular ligation over intramolecular cyclisation by butelase 1. Intermolecular ligation of short peptides followed by head-to-tail cyclisation results in the formation of cyclo-oligomers (Scheme 3A).^48^ Notably, it was highlighted that butelase 1 was not able to recognise the corresponding cyclo-oligomeric peptide products despite the presence of a suitable recognition sequence.^48^ Since AEP catalysis is not known to involve co-factors or other external sources of energy, this observation may be rationalised by conformational stabilisation of the cyclised peptide and an entropic gain from the release of the cleaved recognition peptide motif.

The proximity of the N and C-termini was shown to influence the efficiency of AEP-mediated cyclisation of larger peptide and protein substrates. While butelase 1 was reported to effectively cyclise recombinantly prepared interleukin-1 receptor antagonist (IL-1Ra) and human growth hormone (somatropin), no cyclic proteins were detected when IL1-Ra and somatropin were denatured prior to incubation with the enzyme (Scheme 3B).^37,49^ A similar effect can also be observed in OaAEP1b-mediated protein cyclisation of an intrinsically disordered protein, merozoite surface protein 2 (MSP2), which proceeds in low yield.^39^

### Rate of peptide cyclisation by AEPs

The reported kinetic parameters for peptide cyclisation catalysed by various AEPs have been summarised in Table 1. As investigations have conducted the peptide cyclisation assays under different reaction conditions and substrates, it is difficult to draw direct comparisons. Nevertheless, the catalytic efficiencies of AEPs are relatively high compared to sortase A, a widely used transpeptidase with catalytic efficiency (*k*_cat_/*K*_M_) values ranging from 0.02 to 2.80 × 10^4^ M^−1^ s^−1^ for various engineered variants.^50–52^

**Table 1:** Kinetic parameters of AEP for peptide backbone cyclisation

Enzyme	Origin	Recognition motif	*k*_cat_/s^−1^	*k*_cat_/*K*_M_/10^4^ M^−1^ s^−1^	Assay conditions		Ref.
					Temperature/°C	pH	
Butelase 1	*C. ternatea*	GI-X_*n*_-NHV	26.55	131	42	6.0	23 and 49
OaAEP1b	*O. affinis*	GL-X_*n*_-NGL	0.99	0.68	r.t.	5.0	39
OaAEP1-C247A	*O. affinis*	GL-X_*n*_-NGL	13.9	3.42	37	6.0	10
OaAEP1-C247A	*O. affinis*	GL-X_*n*_-NGL	1.52	1.04	r.t.	6.0	41
OaAEP3	*O. affinis*	GL-X_*n*_-NGL	0.61	33.0	r.t.	5.0	39
OaAEP4	*O. affinis*	GL-X_*n*_-NGL	0.76	98.4	r.t.	5.0	39
OaAEP5	*O. affinis*	GL-X_*n*_-NGL	0.59	24.7	r.t.	5.0	39
Pro-domain-free OaAEP1-C247A	*O. affinis*	GL-X_*n*_-NGL	1.54	0.79	r.t.	6.0	41
xCeAEP1	*C. ensiformis*	GR-X_*n*_-NGL	N.R.	0.58	37	5.0	24
MCoAEP2	*M. cochinchinensis*	GG-X_*n*_-NAL	19.86	62.1	22	6.0	27

With *k*_cat_/*K*_M_ up to 131 × 10^4^ M^−1^ s^−1^, butelase 1 extracted from *C. ternatea* is a highly efficient enzyme for transpeptidation (Table 1).^23,37^ The enzyme was reported to cyclise >95% of linear substrate peptide within 1 h at 37 °C (0.125 μM butelase 1 and 50 μM peptide).^23^ On the other hand, OaAEP1b exhibits a lower *k*_cat_/*K*_M_ than butelase 1.^22^ However, by replacing the gatekeeper (Cys247) near the active site with a smaller Ala residue, the engineered protein was shown to cyclise a model substrate peptide with *k*_cat_/*K*_M_ value of 3.42 × 10^4^ M^−1^ s^−1^ at pH 6.0 and 37 °C.^10^

Subsequently, a suite of AEP ligases from *O. affinis* (OaAEP3, OaAEP4 and OaAEP5) have been identified.^39^ OaAEP4 was reported to cyclise >95% of linear substrate peptide within 30 min at room temperature (0.185 μM AEP and 280 μM peptide) with good tolerance to organic co-solvents (acetone, dimethylformamide and methanol). In directly comparable experimental conditions, OaAEP3, 4 and 5 all demonstrated greater *k*_cat_/*K*_M_ than the prototypic OaAEP1b at pH 5.0 and room temperature. Further differences in the activity of AEPs from *O. affinis* were shown when the peptide cyclisation assay was performed at varying pH values. OaAEP4, the most active among all of the tested AEPs, offered optimal conversion of the linear peptide at pH 4.2 and remained active over a relatively narrow pH range (3.7–5.0). In contrast, OaAEP1b demonstrated optimal activity at pH 5.0 with a broader tolerance to pH compared to OaAEP4 (4.2–7.5). A 50% conversion to the cyclic peptide was detected when linear peptide substrates were incubated with OaAEP1b for 30 min at pH 7.5 (0.528 μM AEP and 280 μM peptide).^39^

## Applications of AEP ligase activity

The growing importance and diverse applications of protein science in contemporary research encompass a range of research topics including protein labelling for the study of post-translational modification;^53–56^ protein-drug conjugation for the production of biopharmaceuticals;^57–59^ peptide ligation for the semi-synthesis of small proteins;^60^ bioconjugation to solid supports for the immobilization of proteins.^61,62^ The ability to facilitate peptide ligation and backbone cyclisation *via* a relatively short substrate recognition sequence, with good catalytic efficiency and tolerance to a broad pH range, highlights the significant potential of AEPs in applications.

### Intramolecular ligation by AEP results in backbone cyclisation

Cyclisation often reduces conformational flexibility and imparts greater thermal and chemical stability.^30,39,49,63^ The joining of a peptide's termini can provide resistance towards degradation by exopeptidases and improve the oral bioavailability of peptide products.^29,30,49,64^ Chemical approaches towards backbone cyclisation, such as native chemical ligation, are limited by strict requirements for N-terminal cysteines (or their surrogates), C-terminal thioesters and denaturing conditions.^63,65,66^ Similarly, the introduction of large split intein domains often impair solubility which limits the versatility of *trans*-protein splicing strategies.^67,68^ Cyclisation *via* small synthetic molecules have been employed to generate diverse libraries of cyclic peptides.^69^ Chemical linkers with two thiol reactive functional groups were reported to bridge across two cysteine residues to afford cyclic peptide species and allow the insertion of non-native motifs.^69^ However, this “chemical bridging” approach exploits the thiol reactivity of cysteine residues, which have limited selectivity when applied to cysteine-rich protein constructs.^69^ Moreover, the termini of bridged cyclic peptides remain unmodified, and thus susceptible to degradation by exopeptidases. The use of peptide ligases offers an alternative approach to peptide cyclisation. With excellent reaction kinetics and a short recognition sequence that is orthogonal to the existing enzymes such as the prolyl oligopeptidase, PCY1,^70^ and transpeptidase, sortase A,^63,71^ the addition of AEPs complements existing strategies.

Peptide backbone cyclisation has been identified as the native function of AEP ligases isolated from plant species which produce cyclic peptides such as *O. affinis* (OaAEP1b),^21,22^ and *C. ternatea* (butelase 1).^23^ In nature, AEP ligases process the linear peptide precursors to afford the cyclic product which are typically around 30 amino acids in length.^64^ Native and engineered AEPs derived from various plants have also been successfully employed to facilitate backbone cyclisation of peptides derived from other plant and non-plant species such as kalata B1;^21–23^ sunflower trypsin inhibitor 1;^23,45,72^ histatin-3;^23^ anti-malarial peptide R1;^39^ MCoTI-II;^27^ AS-48,^72^ and proteins such as GFP;^10,40,49^ somatropin;^49^ MSP2;^39^ p53 binding domain.^73^ Butelase 1 was reported to generate the cyclic antimicrobial peptide θ-defensin and the cyclic conotoxin MrlA with >95% yield in 1 minute at 42 °C and pH 6.0.^45^

**Fig. 6:**
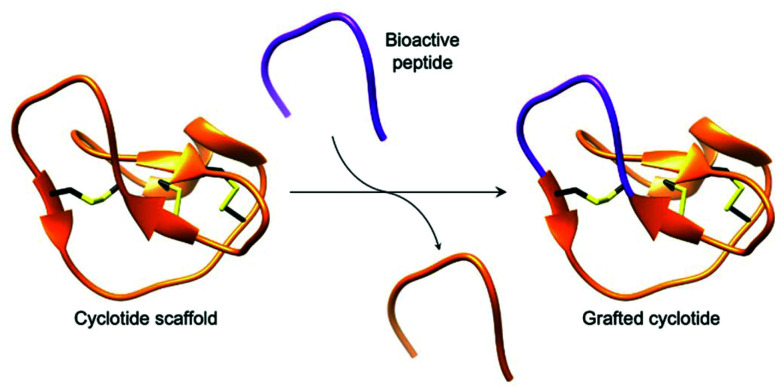
Cartoon representation of a grafted cyclotide. A bioactive peptide (purple) is inserted into a cyclotide backbone, shown here using the structure of MCoTI-II (PDB: 1IB9).

Cyclotides, backbone cyclised and cysteine knotted peptides, have been touted as a potential scaffold for the development of novel therapeutics.^27,29,49^ The emergence of convenient and effective peptide cyclisation methods by AEPs have enabled the synthesis of grafted peptides, whereby bioactive peptides are inserted into cyclotides (Fig. 6).^74^ Exemplified by the cyclisation of MCoSST-01 using MCoAEP2,^27,75^ this approach employs the highly stable cyclic cysteine knot motif from cyclotides as a scaffold to deliver peptide pharmacophores.

Applications of AEPs in protein research were demonstrated by the butelase 1-mediated cyclisation of the p53 binding domain, a potential target for anti-cancer therapeutics. The cyclic protein was reported to aid structural investigations by NMR and X-ray crystallography, methods that may be hindered by conformational flexibility induced line broadening and poor crystal formation, respectively.^73^ Furthermore, greater conformational stability in the cyclic protein was suggested to offer improved ligand binding due to a reduction of the associated entropic penalty.^73^ Protein cyclisation by AEP was also applied to MSP2 from *Plasmodium falciparum*, a 25 kDa protein for malarial vaccine development.^39^ When produced recombinantly, the protein is disordered and its antigenic behaviour no longer represents the native protein.^76–78^ OaAEP1-mediated cyclisation of MSP2 was suggested to reduce disorder and potentially force the protein into adopting a conformation similar to the native antigen.^39^

**Fig. 7:**
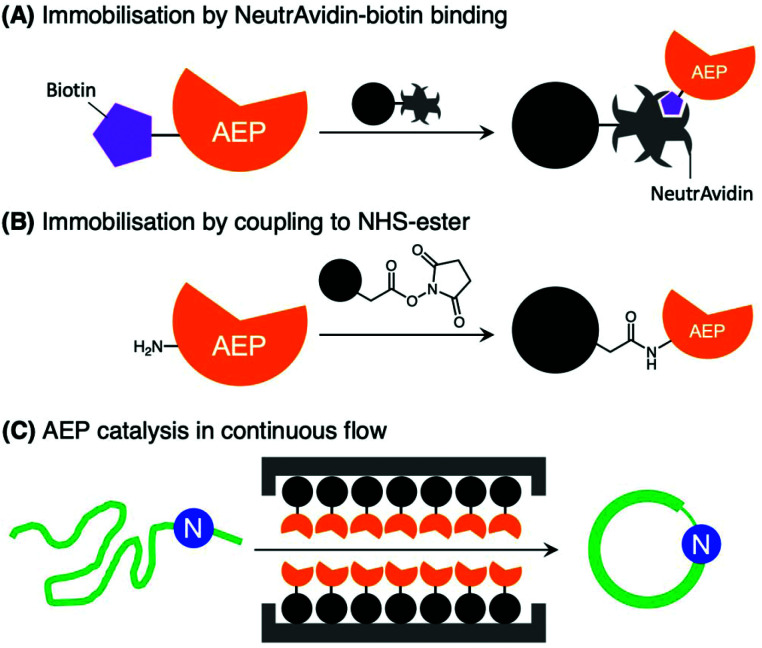
Immobilisation of AEPs on solid support. AEPs, butelase1 and VyPAL2, have been immobilised on agarose beads *via* (A) NeutrAvidin-biotin affinity binding and (B) direct coupling to *N*-hydroxysuccinimide (NHS) ester with primary amines presented by lysine residues. (C) The immobilised AEPs were reported to facilitate ligation of proteins and peptides in continuous flow.

Further developments in AEP-based technology can also be found in a recently published article that explored the effects of enzyme immobilisation (Fig. 7).^72^ The study illustrated that AEPs from *C. ternatea* (butelase 1) and *V. yedoensis* (VyPAL2) which were conjugated to a solid support offered greater stability in storage and repeated use for modifications of polypeptide including labelling and cyclisation. Significantly, immobilisation enabled the use of these enzymes at higher concentrations, and to perform peptide cyclisation reactions in continuous flow.^72^ The findings reported by Hemu *et al.* represent an advance towards adapting AEP catalysis for large scale and industrial applications.^72^

### Intermolecular ligation by AEP for peptide and protein bioconjugation

Naturally occurring proteins commonly feature non-ribosomal modifications. Glycosylation, methylation, and lipidation can influence protein stability, localisation and interactions with other biological components.^54,56,79–81^ Protein modifications are routinely employed to study and engineer proteins beyond their native functions for academic research in addition to medicinal and industrial applications.^55,82,83^ Consequently, the development of novel polypeptide ligation technologies has been a growing theme in biological and organic chemistry research.^51,52,58,84–99^

Currently there is a range of chemical and biological approaches to bioconjugation.^51,52,58,84–99^ Chemical approaches tend to be more affordable, simpler to perform, but can be hindered by poor selectivity or the availability of specific reactive handles.^93,94^ Strategies employing enzymes offer an attractive alternative, whereby site-specific reactions can be catalysed under relatively mild conditions.^95,96^ Transpeptidase from Gram-positive bacteria (sortase A),^51,52,97^ and other enzymes such as subtiligase,^84,98,99^ biotin ligase,^85,86^ transferases^87–89^ and formyl glycine generating enzymes^58,90–92^ have been employed for protein bioconjugation. Excellent kinetic parameters and relatively short recognition sequences indicate that AEP methodologies would be a valuable addition to supplement the existing approaches. AEPs have been employed to modify protein substrates including GFP,^46,47,100^ ubiquitin,^37,46,101^ ompA,^102^ DARPin,^101^ maltose binding proteins,^42,47^ and nanobodies^40^ with a range of synthetic labels including click handles, polyethylene glycol, fluorophores and drug molecules.

**Scheme 4:**
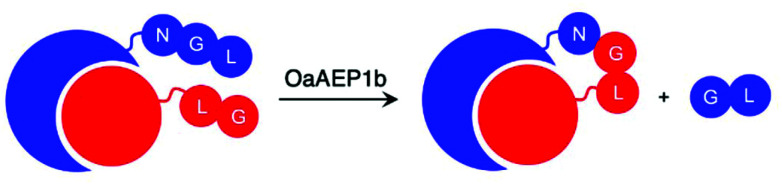
Ligation of self-assembling protein domains by OaAEP1b. Pre-organisation of the ligating substrates bring the reactive sites into close proximity, which was shown to affect AEP catalysis.

Introduction of an isotopically labelled segment into protein backbone by OaAEP1b-mediated ligation was shown to enable NMR studies of maltose binding protein.^47^ The reported method uses a nicked protein, or self-assembling protein domains, to ensure close proximity between the ligating motifs, one of the factors that affects AEP-mediated ligation (Scheme 4).^47,100^ While preparation of labelled proteins by recombinant gene expression is routinely performed,^103–105^ this enzymatic approach offers the ability to selectively label specific regions within the protein. In addition, segmental isotopic labelling of proteins may find other applications such as elucidating protein dynamics.^106–108^

**Scheme 5:**
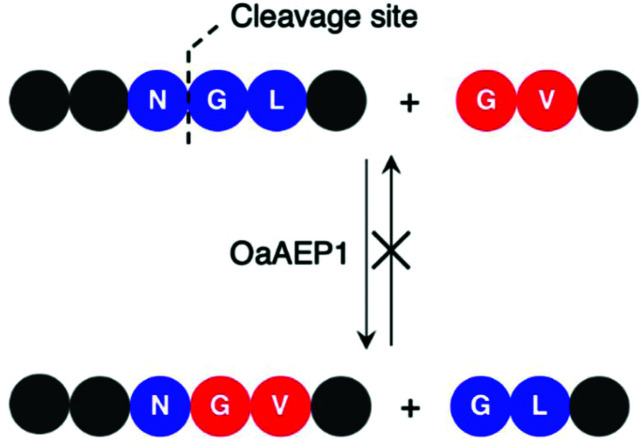
OaAEP1 recognises Val at P2′′, but poorly at P2′. Consequently, this feature in the substrate recognition of OaAEP1 has been exploited to prevent the undesired reverse reaction.

Enzymes commonly target specific substrate recognition motifs defined by the active site. While AEPs have relatively non-specific recognition at certain positions (P1′ and P1′′), changes in the recognition sequence have been shown to influence ligation efficiency.^22,23,27,39,40^ It was reported that, although Gly-Val was accepted by OaAEP1 at the P1′′–P2′′ positions for ligation, the corresponding product (Asn-Gly-Val at P1–P1′–P2′) was poorly recognised for further enzymatic reactions (Scheme 5).^40^ This differential recognition of Val at P2′ *vs.* P2′′ was utilised to construct a protein bearing two different modifications at the N- and C-terminus mediated by a single AEP.^40^ Butelase 1 has also been employed in conjunction with sortase A to modify immunoglobulin molecules (IgG1) with two fluorescent tags (5,6-carboxyfluorescein and AlexaFluor 647) a one-pot dual labelling reaction.^109^ These reports highlight the potential use of AEPs in the semi-synthesis of more complex, multiply labelled protein such as antibody–drug conjugates.

Finally, the reported application of butelase 1 for protein labelling on the surface of live bacteria exemplifies the biocompatibility of enzyme catalysis.^102^ The outer membrane protein A (OmpA) of *Escherichia coli* strain BL21(DE3), modified with the butelase 1 recognition sequence at the C-terminus, was successfully labelled with tags such as 5,6-carboxyfluorescein, biotin and the fluorescent protein mCherry.^102^ The ability to selectively modify proteins on the cell surface can offer an insight towards signalling mechanisms between cells and the extracellular matrix as well as intercellular communications.

## Limitations of using AEP as a biocatalytic tool

### Preparation of recombinant AEP

While the utility of AEPs have been demonstrated through a number of examples, access to active enzyme has hindered the use of AEPs in biocatalytic applications. Butelase 1 was obtained *via* an arduous extraction and purification process from the native host, butterfly pea plant, for initial experiments.^23,37^ Subsequently, procedures for the preparation of recombinant butelase 1 from bacteria (*E. coli*)^36^ and yeast (*P. pastoris*)^73^ were reported. Similarly, the preparation of recombinant OaAEP1b from *E. coli* culture has been reported in a number of studies.^10,22,39,40^

**Fig. 8:**
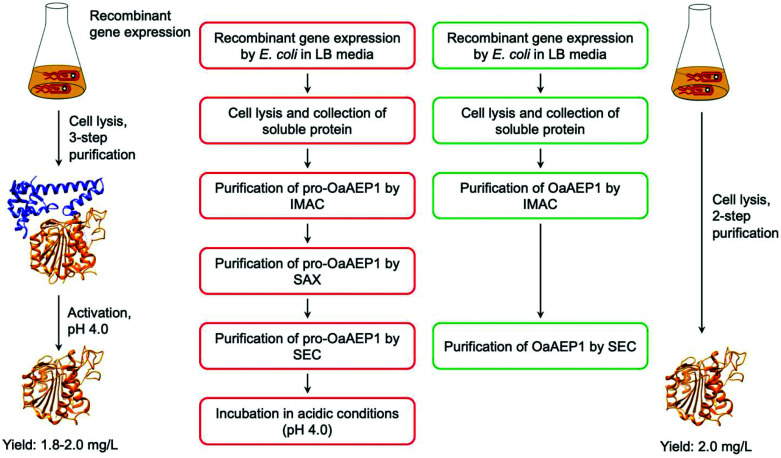
Comparison of procedures for recombinant OaAEP1-C247A. (Red) Recombinant AEP preparation from the pro-enzyme involves multiple purification steps and an incubation period in acidic conditions (pH 4.0). (Green) Recombinant AEP preparation without the pro-domain enabled a streamlined method with less chromatographic steps and does not require activation. (IMAC) Immobilised metal affinity chromatography, (SAX) strong anion exchange and (SEC) size exclusion chromatography. X-ray crystal structure of OaAEP1b (PDB: 5H0I) used here as a representative AEP.

Mimicking the production and maturation of AEPs in nature, the recombinant AEPs are produced as a zymogen which can be activated at low pH (pH 3.6–4.0). The acidic environment simulates the plant vacuole, where active AEPs are known to accumulate in cyclotide-producing plant species.^7,22,24,25^ The gene construct employed to prepare recombinant OaAEP1b encodes for four distinct features: hexahistidine (His_6_) tag, ubiquitin, AEP core domain and pro-domain, producing a fusion construct. Located at the N-terminus, the His_6_ tag facilitates purification by immobilised metal affinity chromatography (IMAC). Between the His_6_ tag and AEP, the ubiquitin sequence has been employed to enhance solubility and expression levels of OaAEP1b and other AEPs.^22,27^ The pro-domain, a C-terminal section of the zymogenic AEP blocks substrate access to the active site, and thus silences enzyme activity. The pro-enzyme was isolated by IMAC, strong anion exchange (SAX) and size exclusion chromatography (SEC). Incubation at low pH (pH 4.0) was required to trigger the auto-proteolytic activation process (Fig. 8). In some studies, the activated AEP was subjected to a further purification step by ion-exchange chromatography.^22,39,42^ The reported yield of the activated OaAEP1b ranged between 1.8 and 2.0 mg L^−1^ from lysogeny-broth (LB) media.^22^ Most of the procedures for preparing recombinant AEPs involved multiple purification steps and an incubation step at low pH. The time consuming and low yielding aspects of the procedure present a considerable limitation to the uptake of AEPs for bioconjugation applications.

To address this issue, gene expression of different constructs have been tested.^41^ A construct based on OaAEP1-C247A that can be expressed without the pro-domain was identified. This has enabled the recombinant preparation of active and soluble AEP which can be isolated in only two chromatographic steps (Fig. 8). The purified enzyme demonstrated activities comparable to those of isolated with a pro-domain (Table 1). Nevertheless, the yield is relatively low (2.0 mg L^−1^) in comparison to most recombinant protein preparation, and hence there remains substantial space for improvement.

### Equilibrium in AEP-mediated peptide ligation

The ligation activity of AEPs have been employed to facilitate intermolecular ligation at either the N or C-terminus of proteins and peptides. While AEP mediated intermolecular ligation has been successfully employed in some cases, it operates with limited efficiency. Similar to other transpeptidases such as sortase A and trypsiligase,^97,110–112^ the challenge in preventing efficient AEP-mediated intermolecular ligation is that the by-product dipeptide (P1′–P2′) acts as a competing nucleophile to reverse the ligation reaction.^40,46^ Water can also potentially serve as nucleophile, leading to hydrolysis. The efficiency of AEP-mediated ligations is affected by the proximity of the ligation partners and the rate of undesired reverse ligation.^37^ Unlike intramolecular cyclisation, the ligating termini are unlikely to be in close proximity. Moreover, the entropic penalty from intermolecular ligation negates any entropic benefits from the release of the cleaved recognition peptide motif. Consequently, a large excess of the labelling peptide nucleophile is required to drive the ligation reaction to completion.^37,40,46,101,109^ The labelling of a nanobody protein using OaAEP1-C247A employed 25 equivalents of a fluorescent peptide label (250 μM), with respect to the protein (10 μM).^40^ This rendered the transpeptidase-mediated ligations uneconomical, especially when expensive or non-commercially available reagents are used (*e.g.* radioactive, isotopic or fluorescent labels).^47,100,113,114^

Attempts to address this limitation were made by exploiting the non-specific substrate recognition nature of AEPs.^23,40,46,47,100,101^ The incorporation of self-assembling protein domains such as nicked-maltose binding protein or designed armadillo repeat protein was the only published approach which enabled effective conjugation without using an excess of labelling reagent.^47,100^ This method employed protein self-assemblies to bring the ligation partners into close proximity, thus improving ligation efficiency (Scheme 4). However, the insertion of a large protein domain into the substrates limits the versatility of this approach. Therefore, a versatile method to enable effective AEP-mediated intermolecular ligation would unlock the tremendous potential of AEPs to a range of applications in protein sciences.

**Scheme 6:**
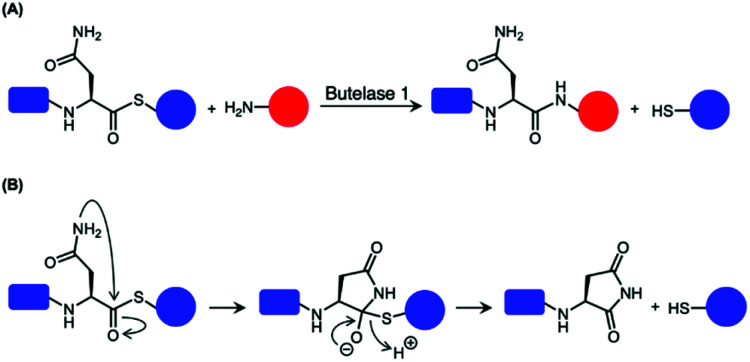
Use of thiodepsipeptide for butelase 1-mediated ligation. (A) Thioester linked Asn is accepted by butelase 1. Peptide ligation generates a peptide by-product with a α-thiol which is not recognised as a nucleophilic peptide substrate. As a result, the reaction was rendered irreversible. (B) Proposed mechanism for the hydrolysis of the unstable thioester.

Butelase 1 have been reported to exhibit considerable reactivity towards synthetic peptide analogues bearing ester and thioester linkages (depsipeptide and thiodepsipeptide, respectively).^46,101^ This was exploited to develop an irreversible butelase 1-mediated ligation strategy (Scheme 6A).^46^ While the depsipeptide approach has been employed to ligate a synthetic peptide to the N-terminus of ubiquitin and green fluorescent protein (GFP) with good yields in 2.5 h, the asparaginyl thiodepsipeptide motif has a short half-life of around 45–75 min at pH 6.5 likely because of its susceptibility to rearrangement (Scheme 6B).^46,65^ Owing to the hydrolysis of thiodepsipeptides, a five-fold excess of this unstable reagent was required to effectively label the target protein substrates.^46^ The use of an unnatural thioester motif further limits the application of this method to the labelling of protein N-termini.^46^

Without using unnatural (thio)depsipeptide motifs, the reverse reaction mediated by OaAEP1 can be prevented by taking advantage of the subtle difference in reactivity between two recognition sequences. Valine was reported to be an amino acid which is recognised at the P2′′ position, but poorly at P2′. Thus, Gly-Val has been used as a recognition sequence on the nucleophilic peptide to generate a product peptide that is not readily hydrolysed by the enzyme (Scheme 5).^40^ Nevertheless, a large excess of the nucleophilic peptide (20-fold excess) was required to obtain the ligated product in good yields.^40^

**Scheme 7:**
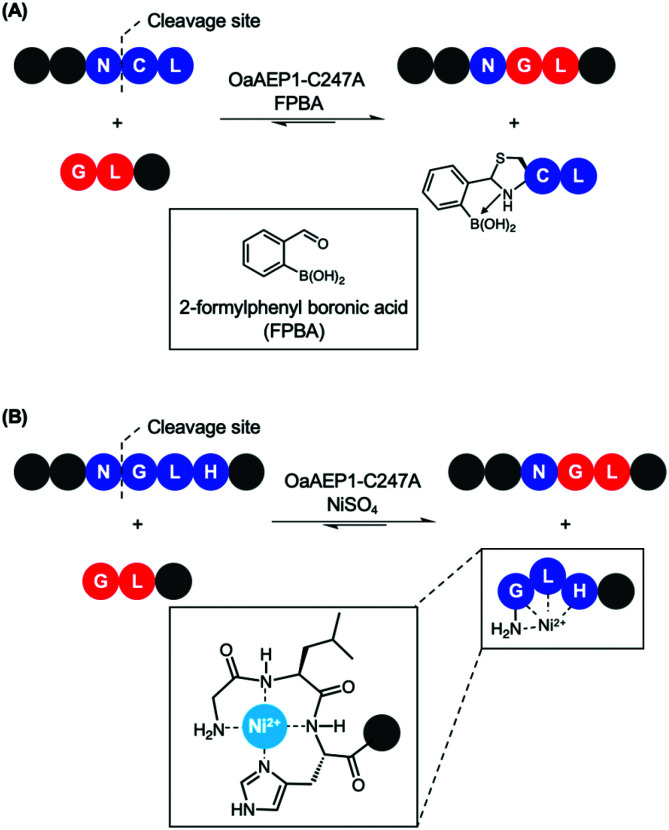
Nucleophile quenching strategies to enable effective peptide ligation by OaAEP1-C247A. (A) FPBA reacts with N-terminal cysteine to thiazolidine derivative to prevent reverse reaction. (B) Divalent Ni^2+^ quenches the nucleophilic peptide by-product generated by OaAEP1-C247A, thus driving the reaction equilibrium towards product formation. The tripeptide motif Gly-Leu-His at the N-terminus coordinates to Ni^2+^.

Recently, reports that coupled AEP catalysis with chemical reactions have been made as an alternative approach to address issues related to reaction equilibrium. In this first report, the recognition Asn-Cys-Leu was recruited to ligate with counterpart carrying a N-terminal Gly-Leu by OaAEP1-C247A. Consequently, the enzymatic reaction can be driven by the addition of 2-formyl phenyl boronic acid (FPBA), which reacts with the released N-terminal cysteine to afford a non-reactive thiazolidine (Scheme 7A).^41^ In another report, a His residue was introduced to the P3′ position. Consequently, the peptide by-product from the OaAEP1-C247A reaction can be sequestered by chelation with a divalent cation such as Ni^2+^ (Scheme 7B). Quenching of the nucleophilic by-product prevents the reverse peptide ligation by OaAEP1-C247A, thus driving the reaction equilibrium towards product formation.^42^ Both of these reports are excellent proof of concepts facilitating protein labelling at the terminus of choice (N or C) with a lowered amount of label. Nevertheless, improvements are still required. Firstly, while the model peptide reactions showed significant improvement by the addition of a quenching agent (+40%), its enhancement was only moderate when applied to protein labelling (+20–30%). Furthermore, the quenching approaches do not address the undesired hydrolysis reaction associated with the protease activity of AEPs. Consequently, the reaction conditions (*e.g.* pH, enzyme concentration, amount of quenching reagent added, addition of reducing agents, reaction time) need to be carefully screened to minimise unwanted hydrolysis reactions. Finally, the reagents employed to quench the nucleophilic by-product may result in off-target interference. For example, reactive aldehydes are commonly employed to introduce protein glycosylation.^92^ Therefore, FPBA may not be applicable towards glycosylated peptide labels. Similarly, the use of Ni^2+^ as a scavenger may also present issues when considering labelling proteins which contain a N-terminal His_6_ tag or utilise divalent metal co-factors such as Fe^2+^.

## Conclusions

Asparaginyl endopeptidases possess a unique combination of features that draw attention from chemical biologists. They are catalytically efficient, and many can be produced using *E. coli* as the recombinant host. AEPs have a short recognition site, which is relatively non-specific at the P1′ position and more stringent at the P2′ site. These features together enable development of various polypeptide modification systems. However, most examples reported in the literature remain at the stage of proof-of-concepts, and great strides are needed in order to put AEPs forward into applications. Crucially, a simplified and high-yielding approach to recombinant AEP is essential. The AEP preparation procedure has recently been simplified to only two chromatographic steps with no incubation at low pH needed. Yet, the yield can be further improved by adapting advanced techniques such as use of an appropriate recombinant host and fermentation. Issues surrounding hydrolysis and reversibility also need to be addressed. The addition of scavengers to the AEP reaction appears to be a straightforward solution to prevent the reverse reaction, but its efficacy on protein labelling require further validation. Nevertheless, with significant advances in the structural biology and enzymology research of AEPs, they are anticipated to be widely adopted biocatalysts in the future to generate polypeptide derivatives that cannot be readily created by existing methods.

## Conflicts of interest

There are no conflicts to declare.
